# Goal Attribution toward Non-Human Objects during Infancy Predicts Imaginary Companion Status during Preschool Years

**DOI:** 10.3389/fpsyg.2016.00221

**Published:** 2016-02-23

**Authors:** Yusuke Moriguchi, Yasuhiro Kanakogi, Naoya Todo, Yuko Okumura, Ikuko Shinohara, Shoji Itakura

**Affiliations:** ^1^Department of School Education, Joetsu University of EducationJoetsu, Japan; ^2^Japan Science and Technology Agency, Precursory Research for Embryonic Science and Technology/SakigakeKawaguchi, Japan; ^3^Graduate School of Education, Kyoto UniversityKyoto, Japan; ^4^National Institute of InformaticsTokyo, Japan; ^5^NTT Communication Science LaboratoriesKyoto, Japan; ^6^National Institute for Educational Policy Research of JapanTokyo, Japan; ^7^Department of Psychology, Graduate School of Letters, Kyoto UniversityKyoto, Japan

**Keywords:** imaginary companion, mentalizing, goal-directed actions, longitudinal study, Bayesian estimation

## Abstract

It has been shown that there is a significant relationship between children's mentalizing skills and creation of an imaginary companion (IC). Theorists have proposed that interaction with an IC may improve mentalizing skills, but it is also possible that children's mentalizing skills affect their creation of an IC. In this longitudinal study, we examined whether goal attribution in infants younger than 1 years old (Time 1) predicted their creation of ICs at 48 months old (Time 2). At Time 1, infants' goal attribution was measured in an action prediction experiment, where infants anticipated three types of action goals: (1) another person's goal-directed action (GH condition); (2) another person's non-goal-directed (BH condition); and (3) a mechanical claw's goal-directed action (MC condition). At Time 2, parents completed questionnaires assessing whether their children had ICs. The path analyses using Bayesian estimation revealed that infants' anticipation in the MC condition, but not in the GH and BH conditions, predicted their later IC status. These results indicate that infants' goal attributions to non-human agents may be a strong predictor of their later IC creation. Early mentalizing skills toward non-human objects may provide children with a basis for their engagement in imaginative play.

## Introduction

Young children enjoy playing with Imaginary Companion (IC) (Bouldin and Pratt, 1999; Taylor, 1999; Gleason et al., 2000; Taylor et al., 2004, 2013; Gleason, 2004a; Moriguchi and Shinohara, 2012; Moriguchi et al., 2015). IC is originally defined as “an invisible character, named and referred to in conversation with other persons or played with directly for a period of time, at least several months, having an air of reality for the child but no apparent objective basis” (Svendsen, 1934). However, recently, personified objects, such as dolls and puppets, are also defined as IC (Taylor, 1999). The present study examined whether this type of children's play was related to an early form of mentalizing, the ability to read the mental states, such as desire, intention, and belief, of other agents (Morton and Frith, 1995; Frith and Frith, 2003).

It has been shown that there is a significant relationship between IC play and mentalizing in young children (Taylor and Carlson, 1997; Roby and Kidd, 2008; Giménez-Dasí et al., 2014), although there is still controversial discussion about the issue (Fernyhough et al., 2007; Davis et al., 2011, 2014). Taylor and Carlson (1997) classified children into high and low fantasy orientation groups based on children's play, such as IC play, and examined whether there were differences in performance on mentalizing tasks (e.g., false belief tasks) between groups. The results revealed that children with high fantasy orientation performed better on mentalizing tasks than those with low fantasy orientation.

Moreover, children with IC are more likely than those without IC to attribute psychological properties to non-human agents. Moriguchi and Shinohara (2012) reported that children with and without IC differed in their attribution of psychological properties (e.g., think) to an invisible agent (but see also, Moriguchi et al., 2015). Wigger et al. (2012) showed that children with IC attributed psychological properties to their IC as well as God. In addition, Tahiroglu (2012) reported that children with IC have a stronger tendency to choose anthropomorphic statements (i.e., attributing psychological properties to non-human objects) compared to those without IC.

Previous research suggests that children with IC have a stronger tendency to attribute psychological properties to other people and non-human objects than those without IC. Theoretically, the dominant view is that IC play improves children's capacity to mentalize. Harris (2000) argued that role play, such as IC play, may improve children's simulation skills, and such skills may facilitate mentalizing. In IC play, children have to simulate how an imaginary agent thinks and feels in a given situation. The simulation process can extend to mentalizing about another person (Roby and Kidd, 2008; Giménez-Dasí et al., 2014) and other non-human objects. The simulation view is supported by recent longitudinal evidence that children's IC status at 3 years of age predicts better performance on false belief tasks (Lillard and Kavanaugh, 2014).

However, an alternative, not mutually exclusive, view is that the early mentalizing skills may be related to the creation of an IC (Dore et al., 2015). The view is inspired by evidence that the frequency of parental mental-state language during infancy predicts children's IC status in their preschool years (Motoshima et al., 2014). According to this view, children who are better at mentalizing may create ICs (Moriguchi and Shinohara, 2012). Children with better mentalizing skills are more likely to attribute psychological properties to other people as well as non-human objects, such as a puppet or a doll (Jipson and Gelman, 2007). Such children can enjoy playing with personified objects or inventing an invisible agent, which may be related to the creation an IC. Indeed, Barrett (2012) argued that mentalizing skills during infancy may be the basis for representing invisible agents. According to this theory, after infants develop the capacity to mentalize an agent, they may use such skills to identify and represent non-human objects as agents. In other words, infants may have a strong sensitivity to the presence of possible agents around them. Barrett suggests that the infants' sensitivity to non-human objects may be the basis for representing and interacting with an invisible agent, such as gods. Thus, the same logic could be applied to representing an IC.

Recent research has shown that infants under 1 year of age are equipped with the precursors of mentalizing, such as goal attribution, and they tend to attribute psychological properties to other people as well as non-human objects (Woodward, 1998; Luo and Baillargeon, 2005; Falck-Ytter et al., 2006; Biro and Leslie, 2007; Csibra, 2008; Yamaguchi et al., 2009; Kanakogi and Itakura, 2011). Importantly, there are substantial individual differences in infants' goal attribution, and some infants are better than others (Sommerville et al., 2005; Aschersleben et al., 2008; Kanakogi and Itakura, 2011). Moreover, the early advantage of goal attribution during infancy predicts better performance in mentalizing tasks at preschool age (e.g., Aschersleben et al., 2008; Yamaguchi et al., 2009).

Given the evidence, the present study focused on infants' goal attribution as a precursor of mentalizing skills, and examined whether infants' goal attributions about other people and non-human objects predicted their IC status at preschool age. In this longitudinal study, children participated in the study when they were younger than 1 years old (Time 1) and when they were 48 months old (Time 2). We chose this age range because a previous study showed that parental mental-state language during infancy predicted children's IC status at preschool age (Motoshima et al., 2014). At Time 1, we used an action prediction task as an index of goal attribution (Kanakogi and Itakura, 2011). In this task, infants were shown videos in which three types of actions were presented: (1) a human's grasping hand was used as the goal-directed action (GH condition); (2) the back of a hand was used as non-goal-directed action (BH condition); and (3) a mechanical claw was used as non-human goal-directed action (MC condition). Using an eye-tracking technique, we measured the time of gaze arrival at the goal relative to the arrival of the observed agents' actions as an index of goal attribution (Falck-Ytter et al., 2006). At 48 months, we examined whether children had an IC based on parental reports.

In the MC condition, we assessed infants' tendency to attribute goal-directedness to non-human objects. Goal attribution to a mechanical claw can be used as an index of goal attribution to non-human, inanimate objects (Hofer et al., 2005). IC play includes children's tendency to attribute psychological properties, such as emotion, goal, and intention, to non-human agents, such as puppet or dolls (i.e., personified object; Taylor, 1999; Harris, 2000). Given the previous evidence, we hypothesized that performance in this condition would be strongly related to children's IC status at Time 2. In addition, we predicted that performance in the GH condition would be moderately related to IC status at Time 2 because this condition assesses the tendency to attribute goal-directedness to another person. In other words, goal attribution in the MC condition during infancy may be directly related to psychological attribution to non-human objects such as puppets, and the tendency may lead to the creation of an IC. However, goal attribution in the GH condition may be indirectly related to psychological attributions to the objects. Given that there might be some overlaps between psychological attributions toward humans and non-humans (Moriguchi and Shinohara, 2012; Wigger et al., 2012), we predicted that goal attributions in the GH condition therefore may be moderately correlated with the creation of an IC. Finally, performance in the BH condition should not predict children's IC status.

## Methods

### Participants

Participants were recruited from a registry of families maintained in the Child Development Lab at Kyoto University. Informed consent was obtained from the children's parents prior to their involvement in the study. The study was conducted in accordance with the principles of the Declaration of Helsinki and the study design was approved by the local ethics committee.

Nineteen children (6 boys and 13 girls) participated in this longitudinal study. The age at the first test (Time 1) varied across participants, and the mean age was 8.5 ± 2.3 months (mean ± standard deviation [SD]; age range = 4.0–10.5 months; 12 girls). All children were 48.0 months at the second test (Time 2). All participants were from middle-class backgrounds. The data from Time 1 were partly reported in Kanakogi and Itakura (2011).

### Research design

Children and their parents participated in the study at Time 1 and Time 2. At Time 1, the study took place in an experiment room at Kyoto University. At Time 2, a questionnaire including questions about ICs was mailed to parents.

### Action prediction experiment

**Figure 1:**
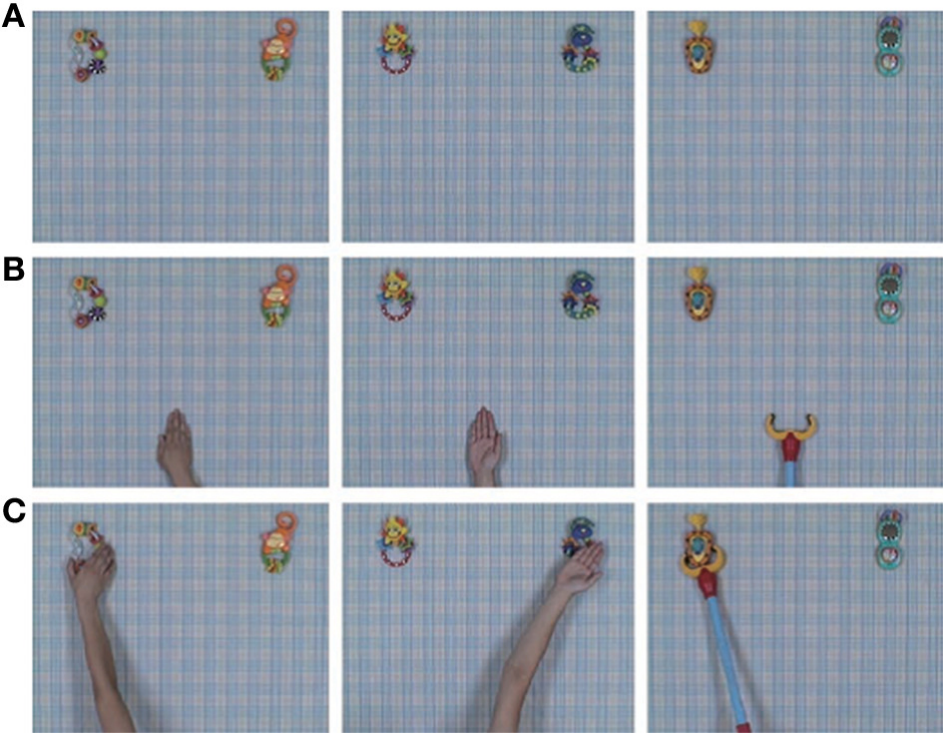
**Selected frames from the video stimuli in each condition**. The conditions are grasping hand (GH, left panels), back of hand (BH, middle panels), and mechanical claw (MC, right panels). **(A)** The agents are out of the frame. **(B)** The agents appear from the bottom of the frame, move upward, and then stop. **(C)** The agents move toward one of two toys, stop at the target toy, and then make contact by grasping (GH, MC) or touching with the back of the hand (BH). We acknowledge Nature Publishing Group for reuse of figures from Kanakogi and Itakura (2011).

The action prediction experiment was the same as in Kanakogi and Itakura (2011), and therefore we described the procedure briefly. A Tobii T60 Eye Tracker (Tobii Technology) was used to record participants' eye movements. Children were shown three types of videos (subtending 19.9° × 16.1° of visual angle) in which agents (human or mechanical claw) reached toward one of two toys at the upper part of the screen from the infant's perspective. There were three types of videos: a grasping hand in the GH condition, the back of the hand in the BH condition, and a mechanical claw in the MC condition (Figure 1). Each video consisted of five components. The hand (or claw) was out of the frame for the first 3 s of the video (Figure 1A). The hand (or claw) then appeared from the bottom of the frame and moved upward (2 s), stopped (1 s) (Figure 1B), moved toward one of the two toys (2 s), and stopped at the target toy (1 s) (Figure 1C). The hand or claw in the GH and MC conditions grasped the target toy, whereas in the BH condition the back of the hand was placed on the target toy. The videos were 9 s in duration, and the duration of each component was controlled across videos. There were six trials in each of the three conditions.

**Figure 2:**
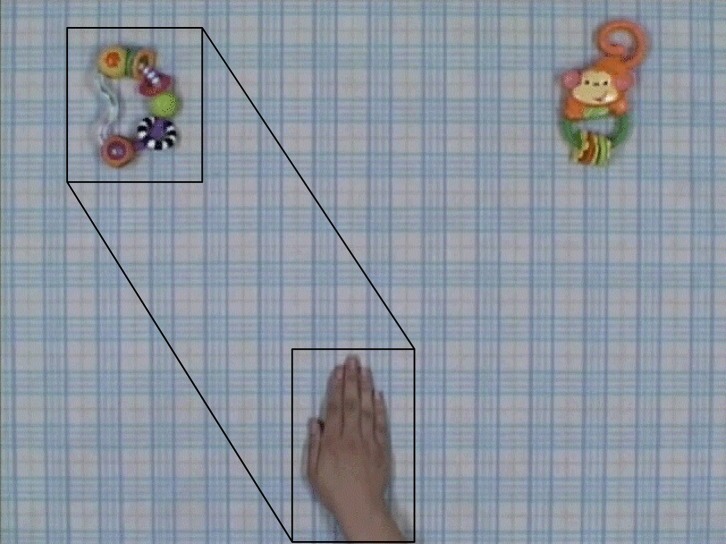
**Analytical examples of stimulus videos and grasping ability**. Example grasping hand condition video. The black rectangles and hexagon represent AOIs within the scene. The upper AOI is the “goal AOI” and encompasses the target object. The lower AOI is the “agent AOI” and encompasses the position where the agent stopped before beginning to move to the target object. The middle AOI is the “trajectory AOI” and encompasses the agent's movement trajectory. We acknowledge Nature Publishing Group for reuse of figures from Kanakogi and Itakura (2011).

All gaze data were analyzed using Tobii's standard statistics package (Tobii Technology). We defined three areas of interest (AOI) (Figure 2): one covering the target object (goal AOI), one covering the position where agents stopped before starting to move toward the target object (agent AOI), and one covering the agent's movement trajectory (trajectory AOI).

The timing of gaze shifts to the goal AOI was compared to the arrival of the agent's action. The arrival of the agent's action was defined as the time when half of the hand (in the GH and BH conditions) or claw (in the MC condition) was located within the goal AOI. If the participant's gaze arrived at the goal AOI before the agent, the trial was regarded as predictive (positive score). In contrast, if the participant's gaze arrived at the goal AOI after the agent arrived at the goal AOI, the trial was not regarded as predictive (negative score).

Data were included in the analyses if the participants' responses met the following criteria for at least two trials in each condition. First, participants had to fixate on both the objects and the agent before the agents had started to move. Second, participants had to fixate on the agent AOI for 200 ms after the agent had moved or fixate on the agent in the trajectory AOI for 200–600 ms while the agent was moving to the target object. Third, they had to fixate on the goal AOI before the video ended. We did not include the first trial of each condition in the analysis because the gaze shift of the first trial is not predictive.

#### Imaginary companions

Parents completed the questionnaire about ICs when the children were 48 months old. A previous study validated the questionnaire in an interview with parents (Motoshima et al., 2014). Moreover, a previous study has shown that parental reports on IC status perfectly matched children's reports (Gleason, 2004b). An IC was defined as a vivid imaginary character that does not actually exist but is treated as real by the child and who the child interacts with during daily activities. Episode examples of both personified-object ICs and invisible ICs were given so that parents understood what was meant by ICs. Then, parents were asked if their child had ICs similar to the examples. If so, parents answered questions about the following: number of ICs; age of the child when the IC appeared and, if relevant, disappeared; age, gender, name, appearance, and personality of the IC; scenes and activities in which the IC was engaged; and child's attitude toward the IC.

Children were regarded as having ICs if parents answered that (1) their child has ICs, (2) the ICs were the same over time, (3) the ICs have names, (4) children and ICs interacted for more than one month.

## Results

### Descriptive results

Mean action prediction scores (SD) in the action prediction experiment at Time 1 were 67.6 (318.6) in the GH condition, −106.6 (362.3) in the BH condition, and −291.7 (289.7) in the MC condition. The SDs were relatively large because participant age varied at Time 1. Statistical analyses are reported below. Next, we report the descriptive IC results. First, we examined the characteristics of each IC group. Four children had both invisible friends and personified objects, 11 children had personified objects, and 4 children did not have any ICs. The number of children with ICs was relatively high compared to previous studies, but the fact that these Japanese children were more likely to have personified objects than invisible friends is consistent with previous studies (Moriguchi and Shinohara, 2012; see also Discussion). Example ICs are presented in Table 1. Three invisible friends were people and one invisible friend was unclear to the parent. In terms of personified objects, all children had personified animal puppets (e.g., bear, monkey, and turtle). Most previous studies did not discriminate children with invisible friend from those with personified objects. However, children show different behaviors to different types of IC. Indeed, research showed that the relationship with invisible friends was more egalitarian whereas the relationship with personified objects was more hierarchical (Gleason et al., 2000). Moreover, children with invisible friends showed better knowledge about another person's social relationship than those with personified object or those without ICs (Gleason, 2002). Thus, there might be qualitative differences between two types of IC. We decided that children scored 1 point for each type of IC (i.e., children who had an invisible friend and personified object scored 2 points). The total IC scores (TO) were used in subsequent analyses. There were more girls in the IC compared to boys, but this difference was not significant (Fisher's exact tests, *p* > 0.10). Because there were no significant differences between groups in terms of sex, sex differences were not analyzed further.

**Table 1:** **Example imaginary companions**.

**Name**	**Description**
Hana-chan	An invisible girl who is cute and has long hair
Panda-chan	A personified panda who is always hungry and likes walking
Me-Me	A personified lamb who is shy
Saru-san	A personified monkey who is like a sibling

### The relationship between action prediction and imaginary companions

#### Path analysis with bayesian estimation

**Figure 3:**
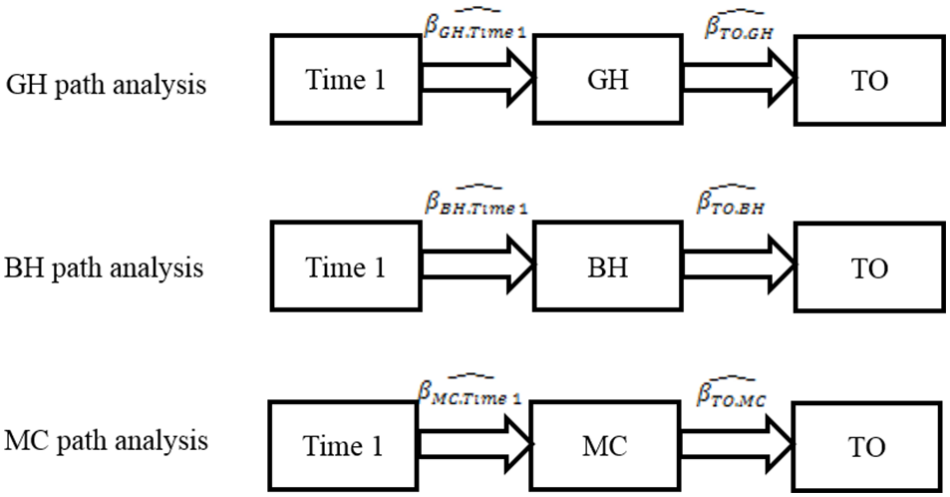
**Path analysis models**.

Our goal was to examine the relationships between action prediction scores in the GH, BH, and MC conditions and total IC scores (TO). However, action prediction scores in each condition were moderately correlated with age (in months). Therefore, we performed three path analyses (one for each condition) to control for age effects (Figure 3).

Because sample size was small (*N* = 19), we analyzed data using Bayesian estimation, more specifically, using the Markov Chain Monte Carlo (MCMC) method (Schoot et al., 2014). We set prior distributions of all parameters as non-informative and used Gibbs sampler as the MCMC algorithm, which are defaults (Muthén and Muthén, 2012). In MCMC, we ran four independent chains with lengths of 100,000, discarded the first half of each chain as burn-in, and used every 50th iteration of the last half to estimate posterior distributions. We checked convergence of each chain using trace plots and auto-correlations. Then, we checked model fits by posterior predictive check and calculated posterior means as point estimates. We also calculated 95% highest density intervals as 95% credibility intervals. We used Mplus ver. 6 for the analyses. In the analysis, we assumed that TO was an ordinal scale.

#### Convergence and model fits

**Figure 4:**
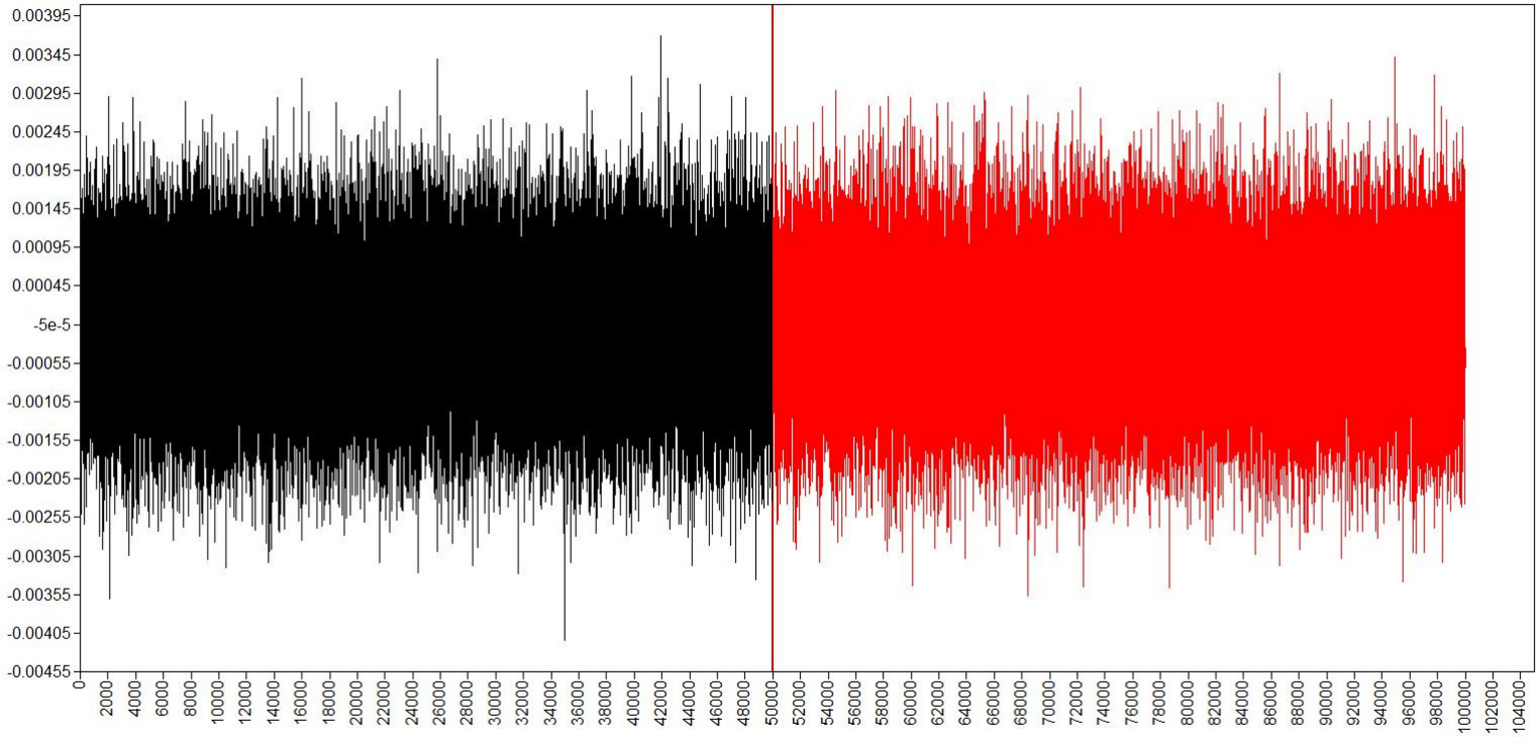
**An example trace plot of all iterations**. This is an example trace plot.

**Figure 5:**
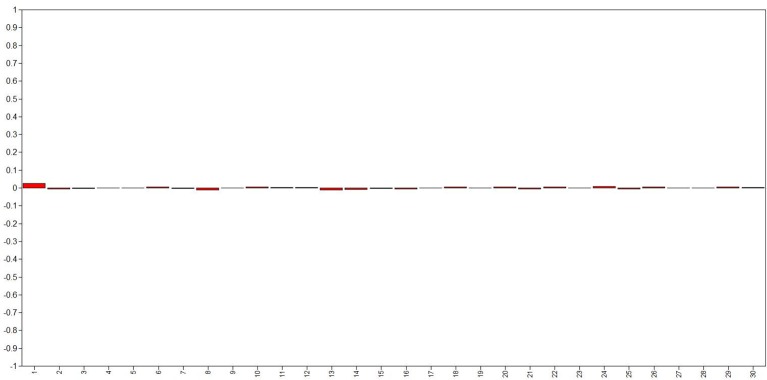
**An example of every 50th iterations' auto-correlation**. This is an example auto-correlation.

First, we checked convergence of each chain using trace plots and auto-correlations. Trace plots and auto-correlations suggested that all chains converged and that estimated posterior distributions were good approximations of true posterior distributions. Examples are shown in Figures 4, 5 which depict trace plots of all iterations and auto-correlations of every 50th iteration, respectively. Next, we checked model fit of the three models using posterior predictive *p*-values. Values around 0.5 indicate that the model fits well. Posterior predictive *p*-values of the three models were 0.550 (GH path analysis), 0.547 (BH path analysis), and 0.522 (MC path analysis). Thus, all models fit the data well.

#### Point estimates and credibility interval

After confirming convergence and model fits, we calculated posterior means and 95% credibility intervals. The results are shown in Table 2. In Table 2, (β_(Y.X)) ^∧^denotes path coefficients from X to Y, σ_Y^∧^2 represents residual variance of Y, μ_Y denotes the intercept of Y, and τ_n represents nth thresholds of TO. In Table 2, path coefficients, (β_(TO.GH))^∧^ and (β_(TO.BH))^∧^, are near zero and their 95% credibility intervals include zero. Therefore, it is likely that GH and BH do not strongly predict TO after controlling for age effects. In contrast, we found a path coefficient (β_(TO.MC))^∧^ of 0.571 and the 95% credibility interval did not include zero. Thus, we confirmed that MC strongly predicted TO even when age effects were controlled.

**Table 2:** **Posterior means and 95% highest density intervals of standardized parameters**.

	**GH path analyses**			**BH path analyses**			**MC path analyses**		
	**Posterior mean (Posterior SD)**	**95% Credibility interval**		**Posterior mean (Posterior SD)**	**95% Credibility interval**		**Posterior mean (Posterior SD)**	**95% Credibility interval**	
		**Lower**	**upper**		**Lower**	**Upper**		**Lower**	**Upper**
$\hat{\beta_{\text{X},\text{ Time }1}}$	0.663 (0.123)	0.415	0.855	0.395 (0.182)	0.037	0.718	0.133 (0.211)	−0.274	0.530
β_TO.X_	−0.032 (0.260)	−0.531	0.463	0.053 (0.273)	−‘0.471	0.573	0.571 (0.201)	0.167	0.894
μ_X_	−2.273 (0.492)	−3.115	−1.296	−1.740 (0.708)	−2.954	−0.290	−1.383 (0.828)	−2.926	0.221
τ_1_	−0.824 (0.321)	−1.440	−0.179	−0.828 (0.322)	−1.473	−0.207	−1.275 (0.316)	−1.901	−0.643
τ_2_	0.813 (0.320)	0.194	1.442	0.801 (0.324)	0.171	1.435	0.295 (0.355)	−0.362	1.010
$\sigma_{\text{X}}^{2}$	0.545 (0.147)	0.283	0.838	0.811 (0.131)	0.574	1.00	0.938 (0.073)	0.782	1.00

*Lower, lower 2.5%; and Upper, upper 2.5%*.

*X in the left column is GH in GH path analysis, BH in BH path analysis, and MC in MC path analysis*.

## Discussion

In the present study, we examined whether infants' ability to predict another person's goal-directed action (GH condition), another person's non-goal-directed action (BH condition), and a mechanical claw's goal-directed action (MC condition) affected their later IC status. The results revealed that goal attributions in the MC condition, but not GH and BH conditions, predicted children's IC status. The results partially support our hypothesis.

To our knowledge, this is the first evidence to show that early mentalizing skills during infancy may affect children's IC status. Previous studies have shown a significant relationship between children's IC status and mentalizing, such as false belief understanding (Taylor and Carlson, 1997; Taylor, 1999; Roby and Kidd, 2008). Although with the exception of a few studies (Lillard and Kavanaugh, 2014) these results are correlational, researchers have assumed that children's interaction with IC may affect the development of mentalizing skills (Harris, 2000; Giménez-Dasí et al., 2014). However, theoretically, it is also possible that children's capacity to mentalize leads to the creations of ICs. Nevertheless, few studies have examined whether early mentalizing skills predict children's IC status.

The present study examined whether infants' mentalzing skills in the GH, BH, and MC conditions predicted their later IC status. The results showed that goal attribution in the MC condition was a predictor of children's IC status. Thus, infants who tended to attribute psychological properties (i.e., goal directedness) to non-human objects may be more likely to create an IC than those who did not. The results may be due to that infants with better goal attribution are more likely to attribute psychological properties to non-human objects, such as a puppet or a doll, and the children can enjoy playing with personified objects or inventing an invisible agent, which may be related to the creation an IC. However, infants' goal attribution in the GH and BH conditions did not predict their later IC status. Because the stimuli in the BH condition were examples of non-goal directed actions, the results are not surprising. However, contrary to our expectation, goal attribution in the GH condition, an index of understanding another person's actions, did not predict children's IC status. These results suggest that goal attribution toward other people and non-human agents may differentially influence children's creation of an IC.

In the literature on goal attribution, two distinctive systems are involved in infants' attributions of goal-directedness to an agent (Johnson et al., 1998, 2001; Luo and Baillargeon, 2005; Biro and Leslie, 2007). One system, a cue-based system, is an innate modular system that identifies goal-directedness (Gergely et al., 1995; Gergely and Csibra, 2003). The modular system is sensitive to behavioral cues, such as self-propelledness (Luo and Baillargeon, 2005), and therefore the system attributes goal-directedness to any agents when infants detect such cues. In contrast, the other system, an experience system, is one that develops an understanding of goal-directedness through experience (Meltzoff, 1995; Woodward, 1998). This system is sensitive to human-like visual properties, such as faces, and therefore the system may attribute goal-directedness to people. Given the literature, it is possible that the former system may be related to the creation of an IC. That is, infants who have a modular system that is sensitive to goal-directedness are more likely to attribute other psychological properties to non-human agents, which may lead to the creation of an IC.

One might argue that the age at Time 1 varies, and such variations may affect both the action prediction at Time 1 and the IC status at Time 2. However, previous research has shown that infants under 1 year of age showed goal attribution, and they tend to attribute goal to other people as well as non-human objects (Woodward, 1998; Luo and Baillargeon, 2005; Falck-Ytter et al., 2006; Biro and Leslie, 2007; Csibra, 2008; Kanakogi and Itakura, 2011). In addition, our analyses controlled the effect of age at Time 1. Nevertheless, we found that infants' goal attributions predicted their later IC status. The results suggest that the age at Time 1 was not a significant factor that may mediate the relationship between goal attribution and IC status. In addition, one might say that the number of children with ICs was relatively high compared to previous studies, and therefore the results in this study may be invalid. However, in a previous study using the same method, nearly half of the parents (*N* = 37) reported that their children had an IC (Motoshima et al., 2014). The results in this study were consistent with the previous results in terms of that children were more likely to have personified objects than invisible friends. Thus, even though the proportion of children with IC was high, we believe that the results in this study were valid.

The results may be consistent with the suggestion by Barrett (2012) that the infants' sensitivity to non-human objects may be the basis for representing and interacting with an invisible agent. On this theory, infants are equipped with skills to identify and represent non-human objects as agents, and such skills may make infants sensitive to the presence of possible agents around them. Such sensitivity, along with the teleological tendency or the concept of the creator, lead to a representation of a god (Barrett, 2012). Thus, in the present study, infants with such sensitivity may be more likely to create non-human agents (ICs) than those without the sensitivity.

Note we are not suggesting that the relationship between IC status and mentalizing can be fully explained by the view that the early mentalizing skills may lead to the creation of an IC. A previous longitudinal study showed that IC status predicted children's mentalizing skills (Lillard and Kavanaugh, 2014). Thus, we propose that the IC and the mentalizing skills are mutually dependent. In other words, it is possible that early mentalizing skills facilitate creation of an IC, which may further improve children's mentalizing skills.

Finally, it is necessary to consider the limitations of the present study. First, the present study did not assess performance in mentalizing tasks at preschool age. Such data would lead to a better understanding of the relationship between mentalizing and IC status. Second, although we conducted analyses with Bayesian estimation, a bigger sample size is desirable. Third, we did not collect data between Time 1 and Time 2, so developmental progress during this period is unknown. Future research should address the relationship between mentalizing and IC status.

## Author contributions

YM developed the study concept. All authors contributed to the study design. Testing and data collection were performed by YK and YO. The data analysis and interpretation were performed by YM and NT under the supervision of SI, YM, and YK drafted the manuscript. NT, YO, IS, and SI provided critical revisions. All authors approved the final version of the manuscript for submission.

### Conflict of interest statement

The authors declare that the research was conducted in the absence of any commercial or financial relationships that could be construed as a potential conflict of interest.
